# An early stage in T4-induced hyperthyroidism is related to systemic oxidative stress but does not influence the pentose cycle in erythrocytes and systemic inflammatory status

**DOI:** 10.20945/2359-3997000000128

**Published:** 2019-04-15

**Authors:** Rayane Brinck Teixeira, Tânia Regina Gattelli Fernandes-Piedras, Adriane Belló-Klein, Cristina Campos Carraro, Alex Sander da Rosa Araujo

**Affiliations:** 1 Universidade Federal do Rio Grande do Sul Departamento de Fisiologia Instituto de Ciências Básicas da Saúde Universidade Federal do Rio Grande do Sul Porto Alegre RS Brasil Laboratório de Fisiologia Cardiovascular, Departamento de Fisiologia, Instituto de Ciências Básicas da Saúde, Universidade Federal do Rio Grande do Sul, Porto Alegre, RS, Brasil

**Keywords:** Hyperthyroidism, reactive oxygen species, sulfhydryl compounds, glucose-6-phosphate dehydrogenase, inflammatory cytokines

## Abstract

**Objective:**

Hyperthyroidism causes many injuries in its target organs and the consequences are reflected systemically. As systemic alterations in hyperthyroidism at earlier stages have received partial attention, this study aimed to investigate systemic redox and inflammatory status at an early stage of T4-induced hyperthyroidism.

**Materials and methods:**

Male Wistar rats were assigned to control and hyperthyroid groups (n = 7/group). The hyperthyroid group received L-thyroxine (12 mg/L) in their drinking water for 14 days whereas control group received only the vehicle. Body weight was measured on the 1st and 14^th^ day of the protocol. On the 14^th^ day, animals were anaesthetized. Blood was then collected from the retro-orbital venous plexus and then the animals were euthanised. The blood was separated into plasma and erythrocytes. Plasma was used to measure ROS levels, sulfhydryl compounds, IL-10, TNF-α and LDH levels; erythrocytes were used for the analysis of thioredoxin reductase activity, glutaredoxin content, and pentose cycle enzymes (total G6PD, G6PD and 6PGD).

**Results:**

Hyperthyroid animals presented body weight gain and final body weight reduction, which was associated with increased ROS levels and decreased sulfhydryl content in plasma. Thioredoxin reductase activity, glutaredoxin content, and pentose cycle enzymes levels in erythrocytes, as well as IL-10, TNF-α and LDH plasma levels were unaltered.

**Conclusion:**

Taken together, our results suggest an impairment in corporal mass associated with systemic oxidative stress at this stage of hyperthyroidism. Meanwhile, the pentose cycle was not influenced and systemic inflammation and tissue damage seem to be absent at this stage of hyperthyroidism.

## INTRODUCTION

Hyperthyroidism is characterised by reduced thyroid-stimulating hormone (TSH) concentration associated with increased thyroid hormones (thyroxine – T4 and/or triiodothyronine – T3) levels, elevated heart rate, and cardiac hypertrophy (1,2). Although many studies have focused on advanced phases, such alterations can be found in even earlier moments in hyperthyroidism. Basset and cols. characterised the thyroid hormone-dependent cardiac hypertrophy at 15 days of hyperthyroidism induction (1). In a similar period at approximately 14 days of T4 administration, Fernandes and cols. showed an association among cardiac hypertrophy and pro-apoptotic signalling in the heart (3).

On the other hand, increased oxidative stress has been demonstrated in different organs, such as the heart, pancreas and liver, at later stages of thyrotoxicosis (2,4,5). Besides the classical markers of redox imbalance, such as lipid peroxidation and protein oxidation, lactate dehydrogenase (LDH) is noted as an important indicator of tissue injury. In addition, LDH seems to increase in oxidised environments (4). Nevertheless, the oxidative stress may provoke an antioxidant response in order to react to eustress state disruption (5). In this sense, thioredoxin and glutaredoxin, important antioxidant proteins, appear increased in stressful situations, such as Grave’s disease and experimental hyperthyroidism (6,7). The oxidised thiol residues of these antioxidant proteins may be recycled through thioredoxin reductase, which reduces the sulfhydryl groups and restores antioxidant protection. In this context, dihydronicotinamide-adenine dinucleotide phosphate (NADPH) is pivotal so that thioredoxin reductase can play on enzymatic function. The NADPH is generated by glucose-6-phosphate dehydrogenase (G^6^PD) and 6-phosphogluconate dehydrogenase (6PGD) in the pentose phosphate pathway (8). However, the pentose phosphate pathway may be inhibited by negative modulators, such as tumour necrosis factor-alpha (TNF-α) (9).

Data in the literature have demonstrated that cellular exposition to TNF-α was previously related to apoptosis and inflammation through the nuclear factor kappa B (NFkB) pathway and caspase-3 activation (10). However, other anti-inflammatory cytokines, such as interleukin 10 (IL-10), may mitigate the inflammation and apoptosis induced by TNF-α (10,11). In this point of view, the IL-10/TNF-α ratio has been used to verify the inflammatory status in different models of disease (11-13). In this context, there is a paucity of information about the role of thyroid hormones on the inflammatory process in earlier stages of hyperthyroidism.

Thus far, the majority of experimental studies investigated advanced stages of hyperthyroidism, and there are not many studies investigating the impact of elevated thyroid hormone levels at earlier stages of the disease. Furthermore, systemic alterations have received reduced attention, and there are few studies describing earlier alterations in plasma and erythrocytes in hyperthyroidism. In this sense, the aim in this study was to investigate the presence of redox and inflammatory alterations at 14 days of the experimental model of T4-induced hyperthyroidism.

## MATERIALS AND METHODS

### Ethical principles

This study was approved by the Ethics Committee for use of animals at the *Universidade Federal do Rio Grande do Sul* (protocol number 24504). All procedures in this study were conducted according to the ethical principles established by the *Universidade Federal do Rio Grande do Sul* and the International Principles Guiding for Biomedical research involving animals from the Council for International Organizations of Medical Science.

### Experimental protocol

Male Wistar rats (166 ± 22 g, 34 days old) were obtained from the central animal house at the *Universidade Federal do Rio Grande do Sul*. Animals were allocated into plastic cages (four animals each) and received water and pelleted food *ad libitum*. They were maintained under standard laboratory conditions (controlled temperature of 21°C, 12 hours light/dark cycle) and were randomly divided into control (n = 7) and hyperthyroid groups (n = 7). Hyperthyroid group animals were submitted to hyperthyroidism induction by administering 0.15 µM L-thyroxine (T4) (12 mg dissolved in 300 µL of 1N NaOH and diluted in the drinking water, final pH = 7, 1 L of final volume) over 14 days, whereas the control group received only the vehicle (300 µL of 1N NaOH diluted in the drinking water, final pH = 7, 1 L of final volume), as previously described in the literature (14). The drinking water of both hyperthyroid and control groups was changed two times per week. The dose of L-thyroxine and the period of treatment were selected based on previous studies (1,3). Based on a previous study, the estimated daily water intake is 35 ml for each animal, which provides a daily dose of approximately 420 µg of L-thyroxine and increases T4 plasma levels by 4-fold after 14 days of treatment (3).

On the 14^th^ day, rats of both the control and the hyperthyroid groups were weighed and anesthetised (ketamine 90 mg/kg and xylazine 10 mg/kg, intraperitoneally). Then 1 ml of blood sample was collected from the retro-orbital venous plexus in heparinised tubes. Each animal was immediately euthanised after blood collection. All the animals were euthanised at the morning. Blood was further centrifuged for 15 minutes at 2415 × g in a refrigerated centrifuge at 4°C (Sorvall RC 5B – Rotor SM 24). Plasma (supernatant) was aliquoted and stored at -80°C for posterior analysis of systemic oxidative stress parameters (total reactive oxygen species [ROS] and sulfhydryl compounds), inflammatory cytokines (plasma levels of IL-10 and TNF-α) and tissue damage (LDH).

The erythrocytes present in the pellet were washed through the addition of saline and centrifugation for 3 minutes at 24640 × *g*. After centrifugation, the supernatant was discarded and the procedure was repeated two more times. Then, the erythrocytes were prepared with an enzyme solution (acetic acid 10 µmol/L and MgCl_2_ 4 mmol/L, 1:50 v./v. of erythrocytes and enzyme solution, respectively), and aliquots were stored at -80°C for posterior analysis of systemic oxidative stress measurements (glutaredoxin content, thioredoxin reductase, G6PD and 6PGD activities). In order to provide reliable data, the protocol described above was repeated twice, obtaining three independent experiments in total, and all results of the present study were obtained from a representative sample of these three independent experiments.

### Oxidative stress measurements

#### Evaluation of the total concentration of ROS by oxidation of 2, 7-dichlorofluorescein (DCFH)

This method of oxidative stress measurement is based on the measurement of the fluorescence produced by the oxidation of DCFH by the ROS present in the plasma sample. Results were expressed as pmol DCF formed/ mg protein (15).

#### Determination of the content of sulfhydryl compounds

Free sulfhydryl compounds in the plasma were assayed by spectrophotometry using the method described by Ellman and modified by Hu and cols. (16,17). The method is based on the reaction of 5,5-dithio-bis(2-nitrobenzoic acid) (DTNB) with thiol groups, resulting in the release of acidic 5-thio-2-nitrobenzoic acid (TNB). Results were expressed as µmol/mg protein.

#### Measurement of thioredoxin reductase activity

The activity of thioredoxin reductase was measured in erythrocyte samples by the reduction of DTNB to TNB, which was detected spectrophotometrically at 412 nm. Data were expressed in nmol/min/mg protein (18).

#### Evaluation of glutaredoxin content

To assess glutaredoxin content in erythrocyte samples, the protocol established by Holmgren & A%slund (19) was utilised. The results were expressed in mmol/mg protein.

#### Activity evaluation of enzymes G6PD and 6PGD

G6PD and 6PGD enzymes determine the production of NADPH, an indicator of redox potential, in order to control the glucose metabolism via the pentose phosphate pathway. Measurement of G6PD was performed in erythrocytes using the method described by Leong & Clark (20). The total activity was expressed in mmol/mg protein. The specific activity of each enzyme was represented as units per mg of protein (U/mg).

## Determination of protein content

Oxidative stress results were normalised by the amount of protein in plasma and erythrocytes, which was quantified by the method of Lowry and cols. (21). Bovine albumin was used as a stock solution in the concentration of 1 mg/ml and diluted in order to obtain a standard curve of albumin.

## Quantification of inflammatory proteins by ELISA and tissue damage by LDH activity in plasma

The quantification of inflammatory proteins in plasma was performed by enzyme-linked immunosorbent assay (ELISA) using IgG ELISA kits for IL-10 (rat IL-10) and TNF-α (rat TNF-α) from Invitrogen Corporation (Camarillo, CA, USA). The results were expressed as pg/mL.

The activity of LDH was measured using a kinetic test with ultraviolet reading based on the method by Wacker & Snodgrass and Amador and cols., which used a Bioliquid assay kit from Laborclin company (Pinhais, PR, Brazil) for plasma samples (22,23). The results were expressed as units of LDH per litre (U/L).

## Statistical analysis

Results are expressed as the mean ± standard deviation. The distribution of data was analysed using the Shapiro-Wilk normality test. Parametric data were tested by Student’s t-test while nonparametric results were tested by independent samples Mann-Whitney U test for comparison between groups. The significance level assumed was 5% (*p* < 0.05). All calculations were performed using SPSS (Statistical Package for Social Sciences, IBM, New York, USA) version 20.0. Graphs were created using GraphPad Prism version 5.00 for Windows (GraphPad Software, San Diego, California, USA).

## RESULTS

### Body weight analysis

**Figure 1:**
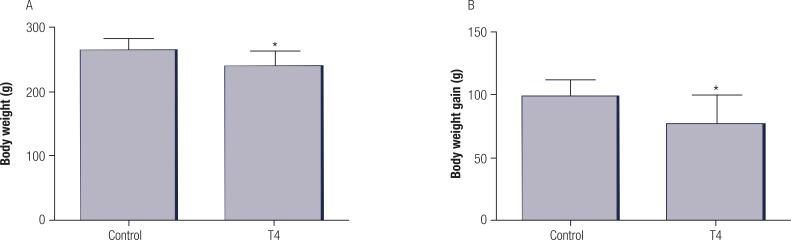
Final body weight (A) and body weight gain (B) values of the control and T4 groups. Values represent mean ± standard deviation (n = 7 animals per group). Data were analysed using the Student’s t-test. *p < 0.05 compared with control group.

Values of final body weight were 10% lower in the hyperthyroid group compared to the control group as shown in Figure 1A. In addition, the body weight gain from the 1^st^ to the 14^th^ day of the experimental protocol was reduced by 22% in the hyperthyroid group as compared to control (Figure 1B).

### Analysis of systemic oxidative stress

**Figure 2:**
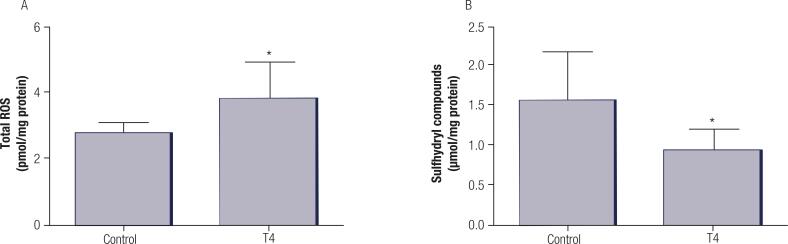
Analysis of total ROS (A) and sulfhydryl compounds levels (B) in the plasma of control and T4 groups. Values represent mean ± standard deviation (n = 7 animals per group). Data were analysed using the Student’s t-test. *p < 0.05 compared with control group.

Results of total ROS in plasma are presented in Figure 2A. The analysis demonstrated increased ROS levels in the hyperthyroid group (36%) compared to the control group. On the other hand, hyperthyroid rats presented reduced levels of sulfhydryl compounds in plasma (39%) as compared to control group (Figure 2B). The activity of thioredoxin reductase, glutaredoxin content, G6PD total activity, isolated activity of G6PD, and 6PGD activity in erythrocytes was similar between groups (Table 1).

**Table 1:** Antioxidant and pentose cycle components measurement

Measurement	Control	T4	P value
Thioredoxin reductase activity (nmol/min/mg protein)	74.19 (49.26; 119.10)	69.27 (60.59; 133.72)	0.902
Glutaredoxin content (mmol/mg protein)	0.55 (0.43; 0.94)	0.61 (0.49; 1.13)	0.710
G6PD total activity (mmol/mg protein)	4.08 ± 1.78	4.49 ± 2.99	0.762
G6PD activity (U/mg protein)	2.80 ± 1.53	3.23 ± 2.15	0.671
6PGD activity (U/mg protein)	1.28 ± 0.52	1.26 ± 0.85	0.943

Parametric data were analysed using the Student’s t-test and expressed as mean ± standard deviation. Nonparametric results were tested using the Mann-Whitney U test and expressed as median and interquartiles (n = 7 animals per group).

### Quantification of inflammatory proteins by ELISA and tissue damage by LDH activity in plasma

**Figure 3:**
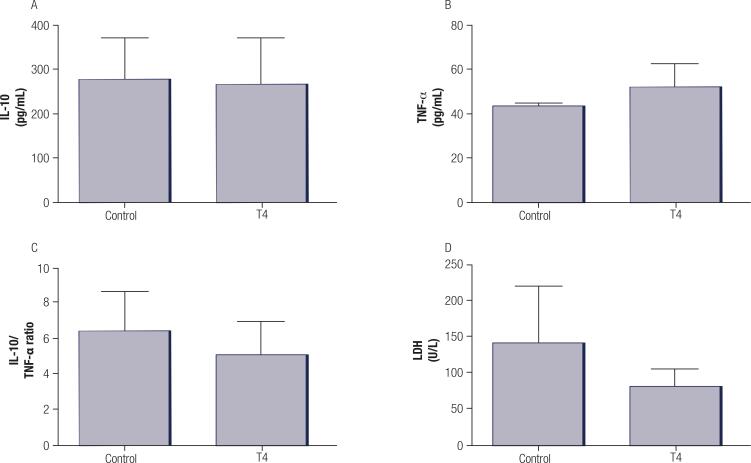
Evaluation of IL-10 levels (A), TNF-α levels (B), IL-10/TNF-α ratio (C), and LDH activity (D) in the plasma of control and T4 group. Values represent mean ± standard deviation (n = 5 -7 animals per group). Data were analysed using the Student’s t-test.

There was no significant difference between groups in terms of IL-10 (Figure 3A), TNF-α levels (Figure 3B), the IL-10/TNF-α ratio (Figure 3C) and LDH levels (Figure 3D).

## DISCUSSION

The major attribute of this study was to demonstrate that systemic oxidative stress plays a more relevant role on hyperthyroidism impairments at this stage (14 days of T4-induction) as compared to the global inflammatory process. The changes in ROS and sulfhydryl compounds in plasma as well as body weight gain alterations suggest not only a systemic distress state, but also reduced body development in hyperthyroid rats. Alterations in inflammatory and tissue damage-related parameters were not found in our study.

The results of body mass reinforce previous studies, which demonstrated a decrease in body weight and body weight gain after 14 days of hyperthyroidism (3,24). This characteristic is a typical consequence in hyperthyroidism, highly related to increases of T4 hormone levels in plasma. Although we didn’t measure T4 levels, a previous study showed 4-fold increase in T4 levels at 14 days of T4 induced hyperthyroidism (3). In this sense, high T4 levels in hyperthyroidism induce a catabolic condition in which there is increased energy expenditure allied to lipolysis and protein degradation (25,26). As a result of weight loss, the hyperthyroidism-imposed inflammatory response may be refractory (27).

Indeed, our results demonstrated that plasma levels of IL-10 and TNF-α, the IL-10/TNF-α ratio and LDH activity remained unaltered at 14 days. This diminished inflammatory reaction may be associated with the reduced mass gain in the hyperthyroid group (28*).* The adipose tissue can produce proinflammatory cytokines, such as TNF-α and IL-6, and activate macrophages therefore establishing an inflammatory environment. Nevertheless, hyperthyroidism provokes lipolysis, reducing adipose tissue and, consequently, downregulates the production of proinflammatory cytokines. Therefore, even in stressful conditions, which are imposed in a state of hyperthyroidism, the weight loss and reduced lipid tissue may attenuate the increase of plasmatic inflammatory protein.

On the other hand, the T4-induced higher demand for energy may augment the mitochondrial ROS formation, which contributes to oxidative stress (29). Indeed, in this experimental model, elevated systemic ROS concentrations were associated with administration of T4 over 14 days. The increased ROS levels in the plasma of the hyperthyroid group could accrue from other organs, representing a systemic response to the disruption of tissue redox homeostasis. This hypothesis is reinforced by the cardiac oxidative status. At one time in this same early period, increased levels of oxygen peroxide were detected and associated with cardiac impaired redox status (3). On the other hand, in hyperthyroidism the elevation of oxidants is associated with an impairment in the capacity of antioxidant defences (2).

In this context, the assessment of total sulfhydryl groups in the plasma provides a wide vision of the thiol-dependent defences, such as glutathione, and cysteine, against free radicals. The low levels of these compounds, as determined in our study, suggest impairment in the antioxidant response (30). No previous study was located that utilised a similar experimental protocol where the sulfhydryl content was investigated in plasma at 14 days. However, previous studies reported decreased sulfhydryl content in patients with advanced stages of hyperthyroidism (31,32). Taken together, increased ROS and decreased sulfhydryl levels in plasma indicate distress status induced by hyperthyroidism at this period.

The oxidative stress associated with depletion in sulfhydryl content could also reflect a decrement of reducing agents, such as NADPH. This coenzyme is produced by the G6PD enzyme, a component of the pentose cycle whose activity is decreased in hyperthyroid patients (33,34). However, this enzymatic parameter in the erythrocytes sample was similar between groups in our study, indicating no alteration in NADPH formation by the pentose cycle at 14 days. In this scenario, it is possible to suggest that the absence of red cell pentose cycle activity elevation in an oxidised environment could compromise the recycling of sulfhydryl groups, since NADPH levels did not increase in parallel to the elevation of ROS concentrations. In this context, the oxidative stress could predominate systemically.

The oxidation of thiol groups may be detrimental to the antioxidant function of thioredoxin reductase and glutaredoxin. In fact, our results showed that thioredoxin and glutaredoxin seem to be unaltered at this stage. However, as previously reported, these proteins could be altered at later stages of hyperthyroidism (2,7,35).

In the earlier stages of hyperthyroidism, the oxidative stress seems to be the preponderant factor, inclining tissues to the adverse adaptation imposed by thyrotoxicosis. This study offered some additional data about the systemic status in the hyperthyroidism, indicating that systemic evaluations could be useful as a tool to monitor the impact of hyperthyroidism on the cardiac remodeling. The lack of data at this time makes it difficult to discuss the findings in this study and reinforced that further studies should be conducted regarding this stage of hyperthyroidism.
